# Correlation of Lateral Cephalogram and Flexible Laryngoscopy with Sleep Study in Obstructive Sleep Apnea

**DOI:** 10.1155/2015/127842

**Published:** 2015-11-24

**Authors:** Anila Narayanan, Bini Faizal

**Affiliations:** Department of ENT, Amrita Institute of Medical Sciences, Amrita Vishwa Vidyapeetham, Kochi, Kerala 682041, India

## Abstract

*Objective*. To study the correlation between lateral cephalogram, flexible laryngoscopy, and sleep study in patients diagnosed with obstructive sleep apnea (OSA).* Background*. Screening tools should be devised for predicting OSA which could be performed on an outpatient basis. With this aim we studied the skeletal and soft tissue characteristics of proven OSA patients.* Methods*. A prospective study was performed in patients diagnosed with obstructive sleep apnea by sleep study. They were evaluated clinically and subjected to lateral cephalometry and nasopharyngolaryngoscopy. The findings were matched to see if they corresponded to AHI of sleep study in severity. An attempt was made to see whether the data predicted the patients who would benefit from oral appliance or surgery as the definitive treatment in indicated cases.* Results*. A retropalatal collapse seen on endoscopy could be equated to the distance from mandibular plane to hyoid (MP-H) of lateral cephalometry and both corresponded to severity of AHI. At the retroglossal region, there was a significant correlation with MP-H, length of the soft palate, and AHI.* Conclusion*. There is significant correlation of lateral cephalogram and awake flexible nasopharyngolaryngoscopy with AHI in OSA. In unison they form an excellent screening tool for snorers.

## 1. Introduction

OSA has received significant attention in sleep medicine. It is considered to be a significant cause of morbidity alerting us to the need of evaluating all snorers. Though polysomnogram is irreplaceable in diagnosing OSA, it makes sense to have simple clinical tools to evaluate OSA patients in the outpatient setting. All patients may not have the time and resources for cone beam CT [1, 2] or drug induced sleep endoscopy, the latest in the management of OSA.

Routine assessment of the patients with OSA includes body mass index, tonsillar grading, thyromental distance, and Modified Mallampati grading. Lateral cephalogram and flexible nasopharyngolaryngoscopy provide multisegmental assessment of airway. Former provides anteroposterior assessment of craniofacial morphology at the level of soft palate and base of tongue. It may not be effective for lateral diametric measurements. Studies have been conducted on cephalometric variables in nonapneic and apneic snorers which can be based as guidelines for recognizing potential candidates.

Flexible nasopharyngolaryngoscopy with Muller's maneuver (awake endoscopy) [3–6] gives a three-dimensional soft tissue assessment of airway. Both the above procedures can be easily done in an outpatient setting. There is no denying that both procedures have better options in the form of cone beam CT and drug induced sleep endoscopy (DISE).

Despite our knowledge that apneic individuals have a collapsing airway, we still have great difficulty in assessing the exact point of collapse during sleep. A combination of lateral cephalometry and awake endoscopy can be a preliminary screening tool to rule out any bony or soft tissue compromise on the airway. DISE and cone beam CT could be the next line of investigation in case of any surgical intervention. This decreases the economic burden on the patient as well as radiation exposure. The level of obstruction in endoscopy may correlate with an altered reading at the corresponding level in lateral cephalometry. It may suggest the efficacy of surgical procedure or suitability of oral appliance in some cases. Conversely, application of these variables in a snorer may provide insights into the probability of OSA in him. However, as suggested by Cavaliere et al. [7] 22.7% of patients with epiglottis related obstruction may go undetected in awake endoscopy. In our study, we analyzed the parameters from lateral cephalometry and awake nasopharyngolaryngoscopy and compared them with AHI of patients with mild, moderate, and severe OSA.

## 2. Materials and Methods

### 2.1. Objectives

To study the correlation of lateral cephalometry and awake flexible nasopharyngolaryngoscopy with Apnea Hypopnea Index of sleep study in patients diagnosed with obstructive sleep apnea.

#### 2.1.1. Study Design

This was a prospective cross-sectional study on patients diagnosed with OSA in a tertiary care centre.

#### 2.1.2. Sample Size and Method of Recruitment

For this study, 70 patients diagnosed with OSA following sleep study, with an AHI of 5 or greater, were included. Patients less than 18 years, who underwent upper airway surgeries, with syndromic anomalies and craniofacial abnormalities like retrognathia, prognathism, and mandibular hypoplasia were excluded. The patients were subsequently divided into 2 groups. group I showed less than 50% of airway collapse on endoscopy and group II had more than 50% collapse.

#### 2.1.3. Data Collection

Patients with symptoms suggestive of OSA underwent sleep study from the Pulmonology Department of our Institute whereby AHI and other parameters like RDI, oxygen saturation, number of apneas, and number of hypopneas were recorded by Apnea Link. OSA is classified as mild (AHI- 5–15), moderate (AHI- 15–30), and severe (AHI- more than 30) depending on the AHI (American Academy of Sleep Medicine).

After routine ENT evaluation, patients underwent flexible nasopharyngolaryngoscopy with Muller's maneuver after adequate nasal preparation with 0.1% Oxymetazoline nasal drops and topical Lignocaine 4%. The following four areas were studied for end expiratory collapse, retropalatal region, retroglossal region, lateral pharyngeal walls, and supraglottic region. Patients were classified into Groups I and II depending on the collapse of airway at these levels.

**Figure 1:**
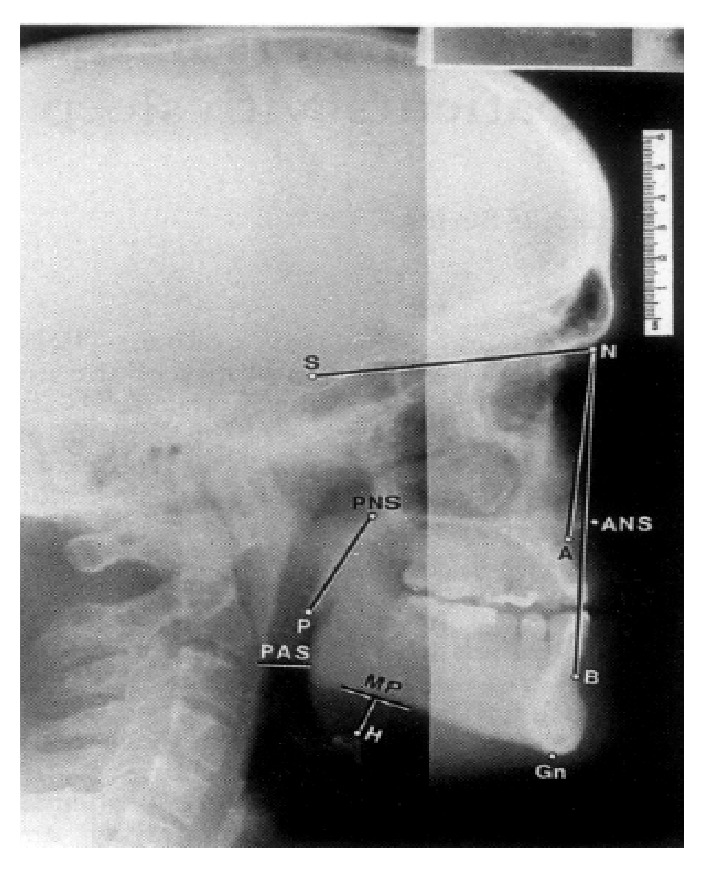
Standard lateral cephalogram with markings for SNA, SNB, PAS, MP-H, and PNS-P. Angle SNA: the angle from sella at the centre of pituitary fossa of sphenoid bone to nasion and the most posterior point on the curve between anterior nasal spine and supradentale of the maxilla. Angle SNB: the angle from sella, nasion, and the point of greatest concavity on the anterior surface of mandibular symphysis. PAS (posterior airway space): the distance from base of tongue to the posterior pharyngeal wall at the level of C2 vertebra. PNS-P (length of the soft palate): the distance from the most posterior point on the sagittal plane of hard palate (PNS) to the tip of the soft palate (P). MP-H (mandibular plane to hyoid bone): measured at the mentum (Me) as the line joining MP (mandibular plane) to H (the most anterosuperior point of the hyoid bone). MP: the line joining (Me) and Go (Gonion, most lateral external point at the junction of the horizontal and ascending ramus of the mandible).

Digitalized lateral cephalometry was taken from the Dental Department of the institute (Sirona; Orthophos XG5). Before the radiograph was taken, a thin layer of barium sulfate paste was applied on the dorsum of the tongue to enhance soft tissue identification. To standardize hyoid position, radiographs were exposed at the end of expiration, in the natural head position with the teeth in light occlusion. The cephalometric variables included in this study were Angle SNA (skull base angle of maxilla), Angle SNB (skull base angle of mandible), PAS (posterior airway space), PNS-P (length of the soft palate), and MP-H (mandibular plane to hyoid bone) (Table 1; Figure 1). These areas were plotted after manual tracing of the lateral cephalograms. The corresponding normal values standardized in our cephalogram are shown in Table 1.

### 2.2. Statistical Analysis

To test the statistical significance of the differences between the two groups, Student's *t*-test was applied. To test the statistical significance of the association of the group with categorical variables Chi square test was done.

## 3. Observations, Results, and Discussion

Our study compared the lateral cephalogram and awake endoscopy with AHI. We could find several significant associations between these investigations.

### 3.1. Correlation of Lateral Cephalometric Variables and Flexible Nasopharyngolaryngoscopy (Awake Endoscopy)

The measurements were taken at the usual sites of collapse, namely, the retropalatal, retroglossal, posterior pharyngeal wall, and supraglottic areas.

#### 3.1.1. Correlation at the Retropalatal Region

**Figure 2:**
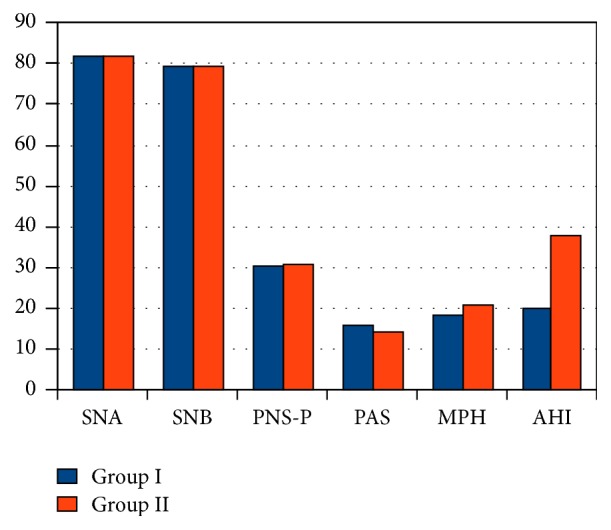
Bar diagram lateral cephalometric variables with retropalatal region.

In group 1 (less than 50% collapse) there were 28 patients and in group 2 (more than 50% collapse) there were 42 patients. MP-H in group 1 was 18.2 mm and in group 2 was 21.1 mm. This value is much less than the normal range of 27 ± 4 mm. This was found to be statistically significant (*p* value <0.001) (Table 2, Figure 2). Though the PAS and PNS-P did show deviation from normal values they were not statistically significant. The findings correlated with the severity of AHI.

#### 3.1.2. Correlation at the Retroglossal Area

**Figure 3:**
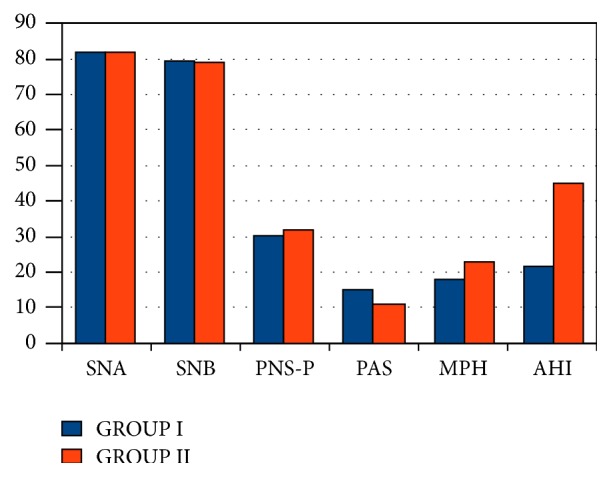
Bar diagram showing association of lateral cephalometric variables with retroglossal region.

Here, group 1 had 43 patients and group 2 had 27 patients. Mean value of PNS-P increased from 30.7 (group 1) to 31.7 in group 2 (normal 17 mm). There was significant statistical association of retroglossal region with MP-H (*p* value <0.001). This correlated well with severity of AHI (Table 4). In our study we could not find any statistical correlation between retroglossal region on flexible nasopharyngolaryngoscopy and the posterior airway space on lateral cephalogram (*p* value 0.934) (Table 3; Figure 3).

#### 3.1.3. Correlation with Lateral Pharyngeal Wall Collapse

In our study, group I had 62 patients and group II had 8 patients and there was statistically significant association noted with lateral pharyngeal wall and MP-H. With high AHI scores there was involvement of lateral pharyngeal wall (Table 4).

#### 3.1.4. Correlation in the Supraglottic Area

The association with supraglottic region could not be done as there were only 2 patients in group II. No statistical association could be made because of the small sample size.

### 3.2. Association of AHI and Findings on Awake Endoscopy

There was significant involvement of retropalatal region with AHI. In mild OSA, 39.28% had retropalatal involvement in group I (Table 5) and only one in group II. In severe OSA the involvement was 25% and 54.76%, respectively.

There was a statistically significant correlation (*p* < 0.001) of AHI with retroglossal collapse (74.07% in group II) (Table 6).

As per Table 7, the lateral pharyngeal wall showed correlation with increasing severity of OSA, though not statistically significant (*p*  0.002). In group I only 12 patients out of 62 had lateral pharyngeal wall collapse. The involvement was significant in moderate and severe OSA with 100% involvement in group II patients.

### 3.3. Association of AHI with Cephalometry (MP-H and PNS-P)

**Figure 4:**
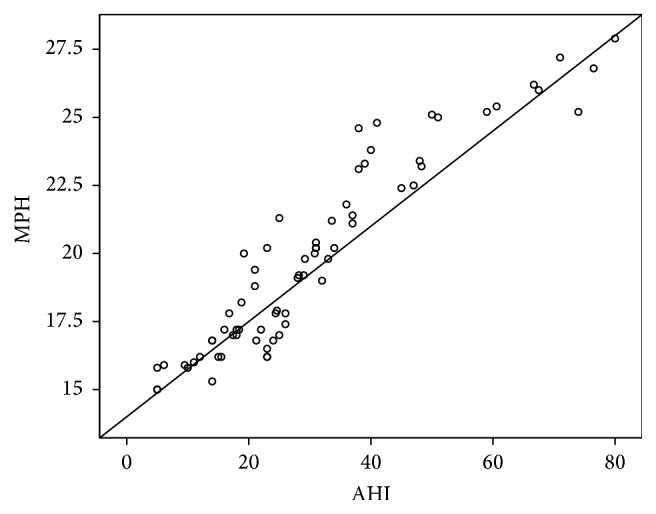
Positive correlation of AHI with MP-H.

**Figure 5:**
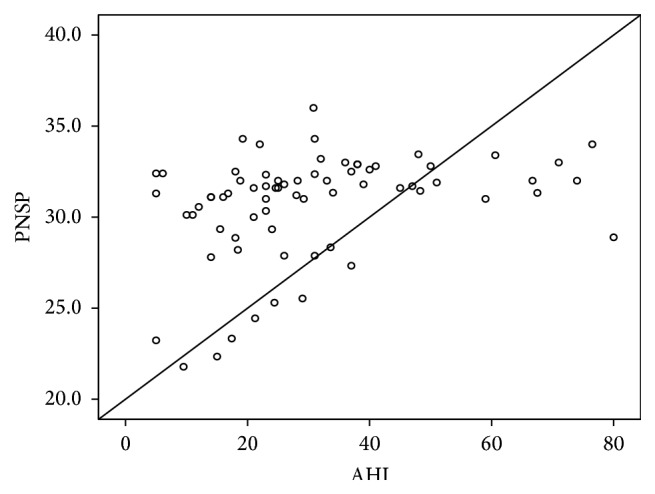
Positive correlation of AHI with PNS-P.

**Figure 6:**
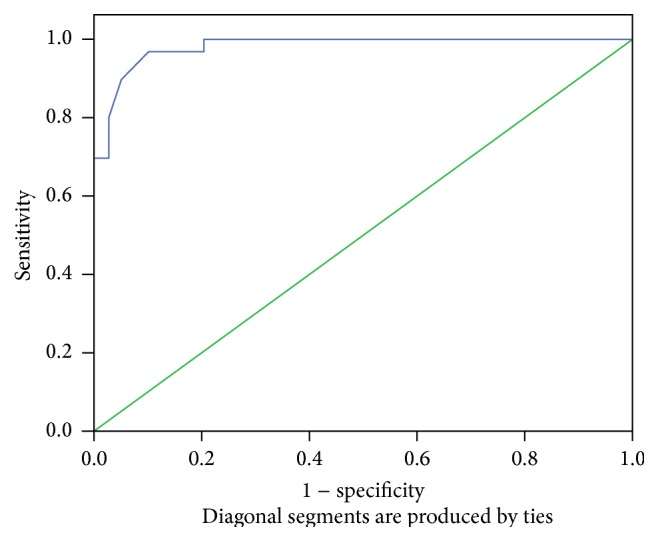
ROC curve for MP-H; area: 0.982.

**Figure 7:**
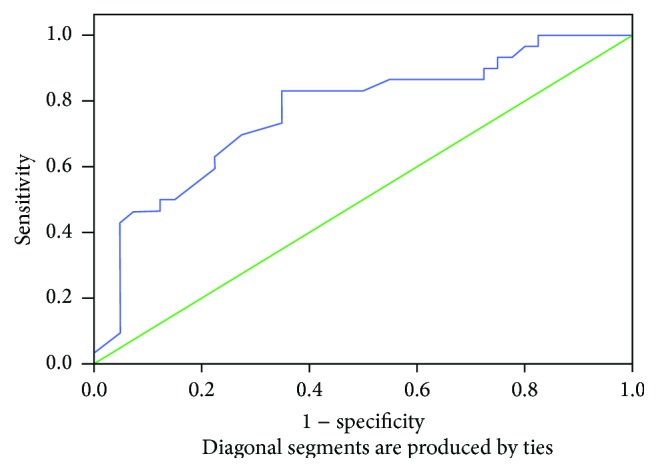
ROC curve area for PNS-P: 0.76.

A significant linear correlation between AHI and MP-H (Figure 4) and PNS-P (Figure 5) was noted (Table 8). Since MP-H was statistically significant in all areas, it was then analyzed by ROC curve to determine a cutoff value and was found to be 21.3 mm (Figure 6). Similarly, the cutoff value of PNS-P was found to be 31.5 mm (Figure 7).

## 4. Discussion

In the retropalatal area, in contrast to our observation, Koo et al.'s study [8] found no significant association between retropalatal region and MP-H. In the latter drug induced sleep endoscopy was performed. Besides, MP-H calculated in our study was manually done with a 0.1 mm calibration which may make it less specific than Jonathan's study where cephalometric software specifically designed for orthodontics was used. We do not have other studies with our background to compare with.

We found a statistically significant association of retroglossal region with PNS-P, MPH, and AHI (Tables 5 and 6). This is in accordance with many studies. This does show that hyoid is at a lower level in OSA patients and the length of soft palate is more. Our PAS was measured at C2 vertebral level. Hence, had we taken a lower level, the posterior airway space and retroglossal area may have shown significant correlation as in Jonathan's study. He measured PAS at multiple vertebral levels. In our study we could not find any statistical correlation between retroglossal region on flexible nasopharyngolaryngoscopy and the posterior airway space on lateral cephalogram (*p* value 0.935).

The relationship of lateral pharyngeal wall collapse in awake endoscopy with cephalometric variables is not possible theoretically since the latter provided only anteroposterior dimensions. But the analysis did show significant correlation with MP-H. This emphasizes interplay of various regions in bony framework and associated changes brought about in the soft tissue structures as a part of a compensatory mechanism. No comparison between these parameters has been done so far.

A significant statistical correlation of lateral pharyngeal wall, retropalatal region, and retroglossal region with AHI (*p* value <0.001) was found in our study (Tables 5, 6, and 7). The observations matched with Koo et al. [8] study though they used DISE. Pang et al. [9] reported that all 3 levels (palatal, lateral pharyngeal wall, and base of tongue) correlated very well with the severity of OSA. Hori et al. [10] reported that there was a significant correlation between the degree of narrowing of retropalatal area and apnea index. As regards to tongue base obstruction, Abdullah et al. [11] noted that there was a significant difference in the frequency and degree of base of tongue collapse in patients with severe OSA. They reported that only 6.9% of patients with mild OSA had more than 50% collapse of the base of the tongue region, as compared to 65.9% of patients with severe grade. In our study, we could find that there were no patients with more than 50% obstruction at the tongue base in the mild group but there were 74.07% of patients in the severe group. However, Ozdas et al. [12] reported that the obstruction and degree of the tongue base had no statistically significant correlation with AHI when examined by Muller's maneuver.

Angles SNA and SNB did not show any correlation in our studies. The results of correlation of AHI and MP-H and PNS-P are similar to Banhiran et al. [13]. There was no statistical significance with length of the palate and AHI which is similar to other studies even though elongated soft palate or excessive tissue in the soft palate is one of the most common causes of snoring and OSA [14–18].

There are anatomical changes which are interrelated explaining that a change in one variable also produces changes in another area in cephalometry or in nasopharyngolaryngoscopy.

Awake endoscopy diagnostically is quite sensitive in assessing the site of collapse in the abovementioned regions except in the supraglottic region. In our study we could only get 2 patients of supraglottic collapse in group II (more than 50% obstruction) which could not be assessed for statistical significance. This is in contradiction to the study by Soares et al. [6] which stated that assessment of awake endoscopy with Muller's maneuver alone is not a good method to detect the pharyngeal collapse site and predict UPPP success. They felt that such patients with retropalatal narrowing had surgical results short of what was expected [13]. Campanini et al. [19] showed, in a retrospective analysis of 250 patients, identical sites of obstruction during awake and sleep endoscopy in only 25% of patients as measured by the Nose Oropharynx Hypopharynx Larynx (NOHL) staging system, introduced by the same author. Soares et al. [20] retrospectively analyzed 53 patients with OSA and compared outpatient department assessment OPDA (endoscopy with and without Muller's maneuver) with DISE to assess the severity of collapse. These did not differ significantly regarding the presence of severe retropalatal collapse but did significantly differ in the incidence of severe retrolingual collapse (DISE 84.9%, OPDA 35.8%).

### 4.1. Limitations of the Study

Since the values in lateral cephalogram were manually traced and calculated to a maximum calibration of 0.1 mm, the accuracy may have been affected. This may explain certain disagreements with a few studies. Smaller sample size might oversubstantiate the findings. Since lateral cephalometry is a two-dimensional investigation, its correlation with a three-dimensional investigation has certain disadvantages on practical aspects. Besides, findings of awake endoscopy involve the patient cooperation and so extrapolation of the findings may be limited.

## 5. Conclusions

There is significant correlation of lateral cephalogram and flexible nasopharyngolaryngoscopy with AHI in OSA.

At the retropalatal region, there was correlation with AHI and MPH of lateral cephalometry.

Retroglossal region showed significant correlation with MP-H, PNS-P, and AHI.

Lateral pharyngeal wall had correlation with MPH and AHI only in partial collapse. It is considered that the involvement of this region is usually secondary to other regions.

The lateral cephalometry parameters and flexiblescopy can predict possibilities of a snorer transforming into an OSA patient. Suitable lifestyle adjustments may prevent such event. Despite the fact that awake endoscopy may miss collapse at the supraglottic region in some cases, it may be used for primary screening of OSA patients. Further, lateral cephalometry can be used as an adjunct to awake endoscopy which captures whole anatomy of the pharyngeal airway with minimum radiation and cost. These should be assessed along with physical parameters for early and appropriate assessment of OSA.

## Figures and Tables

**Table 1:** Showing the normal values of the variables studied.

Lateral cephalogram	Normal values (mm)
SNA	81.1 ± 0.66
SNB	78.3 ± 0.65
MPH	22.5 ± 0.88
PAS	10.4 ± 0.55
PNS-P	39.8 ± 0.57

**Table 2:** Association of retropalatal region on flexible nasopharyngolaryngoscopy with lateral cephalometric variables.

Lateral cephalogram indices	Retropalatal				*p* value
	Group I		Group II		
	<50% obstruction (*n* = 28)		>50% obstruction (*n* = 42)		
	Mean	SD	Mean	SD	
SNA	82	0.7	82	0.7	0.947
SNB	79.5	1.3	79.4	1	0.653
PNSP	30.3	3.7	31	2.3	0.379
PAS	16	4.3	14.1	6.3	0.152
MPH	18.2	2.6	21	3.6	<0.001
AHI	19.9	10.2	38	18.6	<0.001

**Table 3:** Association of retroglossal region on flexible nasopharyngolaryngoscopy with lateral cephalometric variables.

Lateral cephalogram indices	Retroglossal				*p* value
	Group I		Group II		
	<50% obstruction (*n* = 43)		>50% obstruction (*n* = 27)		
	Mean	SD	Mean	SD	
SNA	82.1	0.7	81.8	0.6	0.097
SNB	79.6	1.2	79.2	0.9	0.157
PNSP	30.1	3.3	31.7	1.8	0.011
PAS	14.9	4.2	14.8	7.4	0.934
MPH	18	2.4	22.7	3.1	<0.001
AHI	21.7	10.3	45.2	18.5	<0.001

**Table 4:** Association of lateral pharyngeal wall on flexible nasopharyngolaryngoscopy with lateral cephalometric variables.

Lateral cephalometric indices	Lateral pharyngeal wall			
	Group I		Group II	
	<50% obstruction (*n* = 62)		>50% obstruction (*n* = 8)	
	Mean	SD	Mean	SD
SNA	82.03	0.7	81.6	0.7
SNB	79.4	1.2	79.4	0.9
PNSP	30.6	3	31.5	1.9
PAS	14.6	5.2	16.9	8.4
MPH	19.3	3.3	24.1	2.5

**Table 5:** AHI with retropalatal collapse.

AHI	Retropalatal region		*p* value
	Group I <50% obstruction (*n* = 28)	Group II >50% obstruction (*n* = 42)	
Mild	11 (39.28%)	1 (2.38%)	<0.016
Moderate	10 (35.71%)	18 (42.85%)	
Severe	7 (25.0%)	23 (54.76%)	

**Table 6:** AHI with retroglossal collapse.

AHI	Retroglossal region		*p* value
	Group I <50% obstruction (*n* = 43)	Group II >50% obstruction (*n* = 27)	
Mild	12 (27.9%)		<0.001
Moderate	21 (48.83%)	7 (25.92%)	
Severe	10 (23.25%)	20 (74.07%)	

**Table 7:** AHI with Lateral pharyngeal wall collapse.

AHI	Lateral pharyngeal wall		*p* value
	Group I <50% obstruction (*n* = 62)	Group II >50% obstruction (*n* = 8)	
Mild	12 (19.35%)	0	0.002
Moderate	28 (45.16%)	0	
Severe	22 (35.48%)	8 (100%)	

**Table 8:** Correlations of MPH and PNS-P with AHI.

Variables	*r*	*p* value
MPH × AHI	0.938	<0.001
PNS-P × AHI	0.334	0.005
